# Variations in rates of nosocomial infection among Canadian neonatal intensive care units may be practice-related

**DOI:** 10.1186/1471-2431-5-22

**Published:** 2005-07-08

**Authors:** Khalid Aziz, Douglas D McMillan, Wayne Andrews, Margaret Pendray, Zhenguo Qiu, Stella Karuri, Shoo K Lee, The Canadian Neonatal Network

**Affiliations:** 1 Discipline of Pediatrics, Memorial University of Newfoundland, St. John's NF, Canada; 2 Department of Pediatrics, Dalhousie University, Halifax NS, Canada; 3 Department of Pediatrics, University of British Columbia, Vancouver BC, Canada; 4 Centre for Healthcare Innovation and Improvement, Vancouver BC, Canada; 5 Canadian Neonatal Network Coordinating Center, Vancouver BC, Canada; 6 Discipline of Pediatrics, Memorial University of Newfoundland, St Johns NF, Canada

## Abstract

**Background:**

Nosocomial infection (NI), particularly with positive blood or cerebrospinal fluid bacterial cultures, is a major cause of morbidity in neonatal intensive care units (NICUs). Rates of NI appear to vary substantially between NICUs. The aim of this study was to determine risk factors for NI, as well as the risk-adjusted variations in NI rates among Canadian NICUs.

**Methods:**

From January 1996 to October 1997, data on demographics, intervention, illness severity and NI rates were submitted from 17 Canadian NICUs. Infants admitted at <4 days of age were included. NI was defined as a positive blood or cerebrospinal fluid culture after > 48 hrs in hospital.

**Results:**

765 (23.5%) of 3253 infants <1500 g and 328 (2.5%) of 13228 infants ≥1500 g developed at least one episode of NI. Over 95% of episodes were due to nosocomial bacteremia. Major morbidity was more common amongst those with NI versus those without. Mortality was more strongly associated with NI versus those without for infants ≥1500 g, but not for infants <1500 g. Multiple logistic regression analysis showed that for infants <1500 g, risk factors for NI included gestation <29 weeks, outborn status, increased acuity on day 1, mechanical ventilation and parenteral nutrition. When NICUs were compared for babies <1500 g, the odds ratios for NI ranged from 0.2 (95% confidence interval [CI] 0.1 to 0.4) to 8.6 (95% CI 4.1 to 18.2) when compared to a reference site. This trend persisted after adjustment for risk factors, and was also found in larger babies.

**Conclusion:**

Rates of nosocomial infection in Canadian NICUs vary considerably, even after adjustment for known risk factors. The implication is that this variation is due to differences in clinical practices and therefore may be amenable to interventions that alter practice.

## Background

Hospital acquired (nosocomial) infection in neonatal intensive care units (NICUs) is a significant cause of mortality and morbidity, particularly in very low birth weight (VLBW, <1500 g) babies. VLBW infants when affected by these infections are at increased risk for death, have a longer hospital stay, and utilize more resources than non-affected controls [1]. The 15 participating centres in the National Institute of Child Health and Human Development Neonatal Research Network found that 11% to 32% (mean 21%) of VLBW infants have at least one episode of nosocomial sepsis [2]. Similar variations have been described in other datasets [3,4]. These studies also showed that valid comparisons of rates of nosocomial sepsis between centres require adjustment for risk factors, such as patient demographics and admission illness severity.

Risk-adjustment has also been used to look at variations in rates of both neonatal mortality and morbidity [5]. For example, it has been shown that, after correcting for a number of confounders, Canadian NICUs vary significantly in their rates of death, catheter-related sepsis and intraventricular hemorrhage [6-8]. Each of these authors concluded that differences in outcome may be partly attributable to practice variation. Given the complexity and intensity of care provided to VLBW infants, it is likely that the etiology of acquired infections in NICUs is multifactorial [9], with risk arising from the factors attributable to the host, the organism, the environment and clinical interventions. However, previous studies only examined risk factors for hospital-acquired infections in VLBW infants. It is unclear whether risk factors are different in larger infants with 1500 g or higher birth weights (HBW) admitted to the NICU.

The aims of this study were to examine (a) risk factors for culture-proven hospital acquired infections for VLBW and HBW infants admitted to the NICU, (b) the risks associated with specific clinical interventions in the NICU and (c) the risk-adjusted variations in hospital acquired infection rates among Canadian NICUs.

## Methods

### Study population

The study cohort included all infants admitted to 17 Canadian NICUs prior to 4 days of age. Neonates without a recorded birthweight were excluded. The study cohort was derived from a larger study of 9506 babies admitted to 17 tertiary NICUs in the Canadian Neonatal Network from January 1996 through to October 1997 [10]. These units represent approximately three-quarters of all the tertiary NICU beds in Canada, a nation with >350 000 births annually and almost 30 million people [11,12]. Information about these neonates was prospectively collected by trained abstractors with standardized definitions and operations as part of the larger study of Canadian NICUs [10].

### Variable definitions

For the purpose of this study, nosocomial infection (or NI) was defined as one or more positive single organism blood or CSF culture sampled after 48 h of admission in an infant with clinical suspicion of infection. To differentiate between nosocomial and primary (maternal origin) infections, the infant blood culture isolates were required to be different from maternal isolates or to occur at least 7 days after a treated positive blood culture obtained during the first 48 h of life. CSF samples were obtained when indicated according to local practices and policies. The time period of 2 days was chosen to exclude any neonates born with primary infection. Absence of NI included those infants who were never cultured and those with negative blood and/or CSF cultures. An infection episode was defined as a positive culture occurring at least 7 days after a previous treated culture or if the culture isolates were different from the previous culture [13]. We defined nosocomial infections (NI) in this way because blood and CSF are normally sterile, thus a positive culture from one of these sites in an unwell baby is likely to be of serious clinical significance. Additionally, from a practical perspective, these data are well defined and readily abstracted from patient charts.

Other study definitions were in accordance with the Canadian Neonatal Network Data Abstractor's Manual [14]: gestational age was defined as the attending obstetrician's best estimate based on obstetric history and examination, and prenatal ultrasound, except where this was not available or the postnatal physical assessment disagreed with obstetrical estimate by greater than 2 weeks. In that case, the pediatric estimate of gestational age was used instead. Small for gestational age meant being born below the 3^rd ^percentile for weight (corrected for gestation using growth charts developed by Whitfield et al [15]). Major congenital and chromosomal anomalies were specified in the Abstractor's Manual. Outborn infants were those born at a different hospital from the admitting NICU. The Score for Neonatal Acute Physiology, Version II (SNAP-II) was collected on day 1 as a measure of patient acuity on the day of admission. The SNAP-II is a validated score [16] using 6 empirically weighted physiological measurements made during the first 12 hours after admission to the NICU. To be consistent with previous reports, SNAP-II score was categorized as 0 to 9, 10 to 19, 20 to 29 and greater or equal to 30. Therapy related risk factors were extracted from day-1 and day-3 evaluations, using a subset of relevant variables from the Neonatal Therapeutic Intensity Scoring System (NTISS) [17]. The subset of variables included the use of assisted ventilation, peripheral intravenous (IV) access, central venous access, arterial line access, use of antibiotics (day 1 or 3), gavage feeding, intravenous amino acids, and intravenous fat emulsion.

### Data analysis

Univariate and bivariate analyses using SPSS (Chicago, Illinois) were used to describe the study population and to explore the relationships between baseline population risks (gestational age, birth weight, gender, Apgar score at 5 minutes, small for gestational age, use of antenatal corticosteroids, multiple births, outborn status, caesarean section, maternal hypertension, congenital anomalies, admission illness severity) and NI. Chi-square test was used to compare mortality and morbidity among infants with and without NI, for infants with birth weight less than 1500 g and 1500 g or more.

We used a multivariable logistic regression model to develop a risk adjustment model for NI. The outcome variable was NI and independent variables were baseline population risks significantly correlated with NI on bivariate analysis. Correlation matrix was used to examine correlation between variables. The full model was created by adding population risk factors, then SNAP-II score, and finally therapy-related factors – variables were considered to be included if their p-value was <0.10. Backward and forward selections from the full model were applied to help determine the parsimonious model. Finally, centres were compared using adjusted odds ratios and 95% confidence intervals derived from the multivariable logistic regression analysis, using a reference centre (the hospital with the median incidence of nosocomial infection). Sensitivity analyses were conducted by excluding outlier sites with large confidence intervals.

### Institutional review

This study was part of a larger study surveying morbidities and mortality in Canada, for which ethics approval for anonymous collation of data was obtained at each individual centre.

## Results

### Patient population and nosocomial infection episodes

16538 infants in the dataset met entry criteria (age less than 4 days on admission). 765 (23.5%) of 3253 VLBW infants, and 329 (2.5%) of 13244 HBW infants (41 were missing birth weight and were excluded), developed at least one episode of NI. Among VLBW infants who developed NI, 78.7% had only 1 episode, 16.2% had 2 episodes, and 5.1% had 3 or more episodes of NI, whereas among HBW infants, 87.9% had 1 episode, 9.3% had 2 episodes, and 2.8% had 3 or more episodes of NI. The median onset of the first infection from day of admission was at 19 days for VLBW infants and 15 days for HBW infants.

### Organisms and antibiotics

Among infants with the first episode of NI, the incidence of CSF culture positive infections was 3.7% in VLBW infants, and 2.8% in HBW infants. The most common organism obtained from blood culture was coagulase negative Staphylococcus (72% of isolates in VLBW, and 57% in HBW). The same was true of CSF isolates (68% and 41% respectively). Gram-negative organisms were the next most common pathogen in VLBW, whereas Gram positives, particularly group B Streptococcus, was the next most prevalent in HBW.

Ninety percent of VLBW infants and 73.3% of HBW infants received at least one course of antibiotics in the NICU on either day 1 and / or day 3, as recorded by NTISS

### Infant characteristics and therapy related factors

*Among VLBW infants*, bivariate analysis showed that infants with NI were more likely to be outborn, delivered by vaginal birth, and have 5 minute Apgar score <7, but less likely to be SGA (see Table 1). Infants with NI also had significantly lower birth weight and gestational age, and higher mean day 1 SNAP-II score, than those without NI. A number of therapy related risk factors were significantly associated with NI: these included use of assisted ventilation, peripheral IV access, central venous access, arterial line access, use of antibiotics (on day 1 and/or day 3), gavage feeding, IV amino acid, and IV fat. *For HBW infants*, NI was associated with similar infant characteristics, but also with congenital anomalies, 5 minute Apgar score <7, and higher SNAP-II score. *Correlation between variables: *NI has significant correlation with all variables identified except for antibiotic use. However, when stratified by birth weight, antibiotic use has significant correlation with NI for HBW infants. The Spearman correlation coefficient between CVL and parenteral nutrition is 0.26, which is significant. Furthermore, a cross-tabulation of NI by CVL controlling for IV parenteral nutrition (TPN) indicates significant correlation beween NI and CVL and a contingency coefficient of 0.1 for either TPN present or absent. Therefore, there is no evidence that the presence of TPN or CVL in the models renders the other redundant.

**Table 1:** Characteristics of infants with and without nosocomial infections (NI), for birth weights less than 1500 g and 1500 g or more (asterisks indicate significant difference at p < 0.05 level between infants with and without NI in each birthweight group)

**Birth weight**	**Less than 1500 g**		**1500 g or more**	
**Characterististics**	**With NI **(n = 765)	**Without NI **(n = 2488)	**With NI **(n = 329)	**Without NI **(n = 13244)

Gestation (mean weeks ± sd)	27.2 +/- 2.3	28.8 +/- 2.8*	35.8 +/- 3.6	36.3 +/- 3.2*
Birth weight (mean gm ± sd)	957 +/- 259	1010 +/- 267*	2611 +/- 806	2758 +/- 796*
Day 1 SNAP-II (mean ± sd)	18.2 +/- 12.5	14.1 +/- 13.0*	11.9 +/- 12.5	4.9 +/- 8.5*
Small for gestational age (%)	9.7	12.6*	2.8	2.7
Antenatal steroids (%)	71.4	68.2	21.1	20.4
Multiple birth (%)	26.7	28.5	7.9	12.5*
Outborn (%)	24.6	12.7*	59.9	23.1*
Male (%)	55.3	51.8	59.9	58.1
Cesarean delivery (%)	47.5	55.2*	38.9	36.3
Maternal hypertension (%)	18.1	19.4	10.4	11.6
Apgar @ 5 mins < 7 (%)	26.9	20.0*	21.4	11.9*
Congenital Anomalies (%)	10.6	7.9*	48.6	10.9*

### Mortality and morbidity

Mortality was more strongly associated with the NI group compared to the group without NI for HBW infants, but not for VLBW infants. Major morbidities (necrotizing enterocolitis, chronic lung disease at 36 weeks post-menstrual age, severe intraventricular hemorrhage/periventricular lesions, and severe stages (3 and 4) of retinopathy of prematurity) were more common in the NI group (Table 2).

**Table 2:** Mortality and morbidity among infants with and without nosocomial infections, for infants with birth weight less than 1500 g and 1500 g or more (asterisks indicate significant difference at p < 0.05 level between infants with and without nosocomial infections within each birth weight group).

**Birthweight**	**Less than 1500 g**		**1500 g or more**	
**Outcome (%)**	**With NI **(n = 765)	**Without NI **(n = 2488)	**With NI **(n = 329)	**Without NI **(n = 12915)

Mortality	8.7	8.6	8.5	1.3*
Necrotizing enterocolitis	13.4	4.4*	8.8	0.4*
Chronic lung disease	38.7	18.5*	25.7	4.4*
Severe intraventricular hemorrhage	10.5	8.2*	5.3	2.8*
Severe retinopathy of prematurity	14.6	8.1*	0.0	0.0
Survival without major morbidity	46.3	69.5*	74.8	96.0*

### Risk factors predictive of nosocomial infection on multivariable logistic regression

*In VLBW infants*, factors associated with NI in the multivariable logistic model were: gestational age, use of assisted ventilation and IV fat and, and outborn status (see table 3). Ninety-nine percent of infants receiving IV fat also received IV amino acid, so these interventions were combined. SNAP-II scores were combined into ≥10 and 0–9 (the reference) groups because the parameters for the higher score groups [10–19, 20–29, > = 30) appeared to be similar. Compared to infants with a gestational age of greater than 28 weeks, lower gestation was increasingly more likely to be associated with NI. SNAP-II score ≥10 was of borderline significance.

**Table 3:** Risk factors associated with nosocomial infection on multivariate analysis, for infants with birth weight less than 1500 g and 1500 g or more

**Birth weight**	**Less than 1500 g Odds Ratio (95% confidence intervals)**	**1500 g or more Odds Ratios (95% confidence intervals)**
Gestation ≤24 weeks	2.7 (1.4 – 5.3)	4.0 (0.5 – 32.7)
Gestation 25–28 weeks	2.2 (1.2 – 4.1)	6.1 (1.6 – 23.1)
Gestation 29–32 weeks	1.1 (0.6 – 2.0)	1.9 (1.3 – 2.6)
SNAP II ≥10	1.2 (1.0 – 1.6)	1.0 (0.8 – 1.3)
Peripheral intravenous line	0.9 (0.7 – 1.1)	1.9 (1.1 – 3.2)
Central venous Line	0.9 (0.8 – 1.2)	1.6 (1.2 -2.1)
Assisted ventilation	1.5 (1.1 – 2.0)	2.9 (1.9 – 4.6)
Parenteral nutrition	3.9 (3.0 – 5.2)	5.1 (3.8 – 6.9)
Outborn status	2.0 (1.6 – 2.5)	1.9 (1.4 – 2.6)
Use of antibiotics	0.9 (0.4 – 2.3)	2.9 (1.6 – 5.0)
Congenital anomalies	1.2 (0.9 – 1.6)	2.9 (2.2 – 3.8)

*In HBW infants*, factors associated (p < 0.05) with NI in the multivariable logistic model were: gestational age, use of central venous access, peripheral intravenous access, and use of IV fat or IV amino acid, assisted ventilation, outborn status, use of antibiotics and presence of congenital anomalies. Again, IV fat and IV amino acid could be used interchangeably in the model. Infants who received parenteral nutrition were more likely to have NI. Central venous access was associated with higher risk for NI, as was use of peripheral IV access.

### Variations in nosocomial infection rates among NICUs: site comparisons

**Figure 1:**
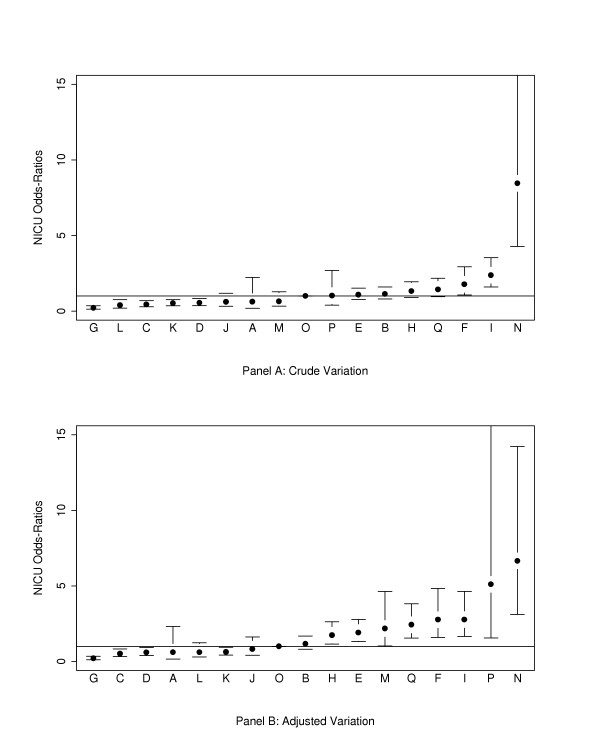
Crude and risk adjusted odds ratios of nosocomial infection rates by hospital for infants <1500 g birth weight (point estimates and 95% confidence interval limits shown). Adjustment is for gestational age, admission day SNAP-II score, and outborn status. Site O, the site closest to the median risk, was used as the reference site.

*For VLBW infants*, cross-tabulation of site and NI showed significant variation between sites (chi-square = 248.7, df = 16, P < 0.001). Crude rates of NI ranged from 6.7% to 74.5% of infants having at least one episode of NI. The crude incidences of NI for 8 hospitals were significantly (p < 0.05) different from the reference hospital (site O) (Figure 1). Site variations persisted after adjustment for baseline patient risk factors (gestational age, admission day SNAP-II score, outborn status) using multivariate logistic regression analysis (Figure 1), with the highest odds ratio being 8.6 (95% confidence interval (CI) 4.1 to 18.2), and the lowest 0.2 (95% CI 0.1 to 0.4) when compared to the reference site (Figure 1).

**Figure 2:**
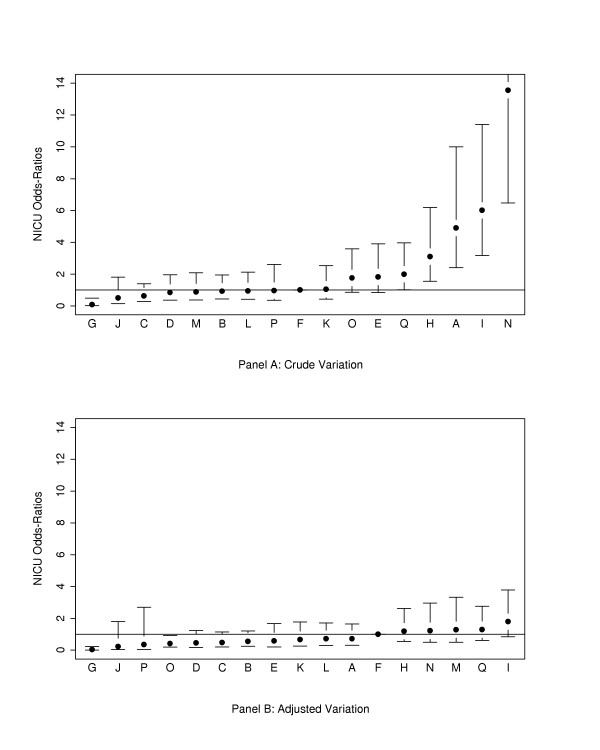
Crude and risk adjusted odds ratios of nosocomial infection rates by hospital for infants ≥1500 g birth weight (point estimates and 95% confidence interval limits shown). Adjustment is for gestational age, admission day SNAP-II score, and outborn status. Site O, the site closest to the median risk, was used as the reference site.

*For HBW infants*, significant variation was also present between sites (chi-square = 360.0, df = 16, P < 0.001). Crude rates of NI ranged from 0.1% to 17.0 %. When only site variables were in the regression model, 6 hospitals had significantly different (p < 0.05) NI rates from the reference hospital (F). Significant site differences largely corrected after adjustment for baseline population risks (gestational age, admission day SNAP-II score, outborn status) (Figure 2).

**Figure 3:**
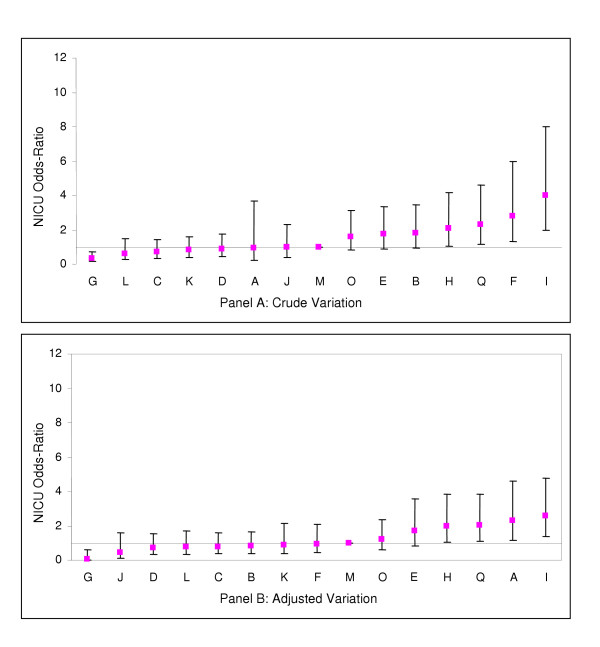
Crude and risk adjusted odds ratios of nosocomial infection rates by hospital (with sites P and N excluded) for infants <1500 g birth weight (point estimates and 95% confidence interval limits shown). Adjustment is for gestational age, admission day SNAP-II score, and outborn status.

**Figure 4:**
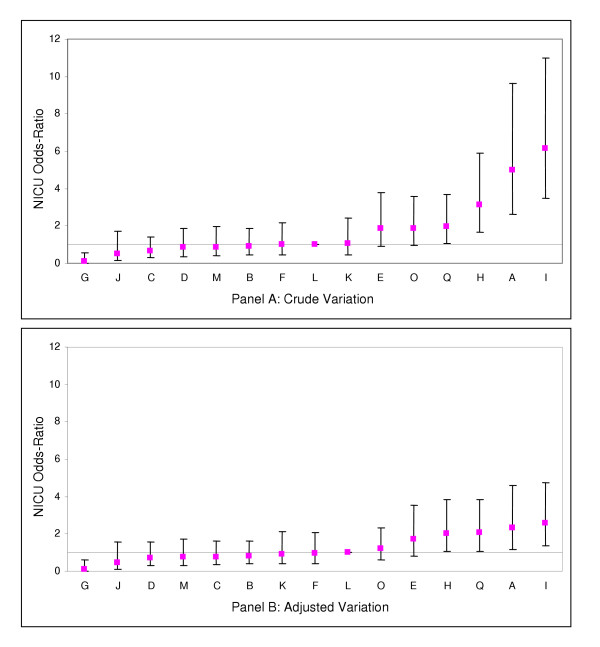
Crude and risk adjusted odds ratios of nosocomial infection rates by hospital (with sites P and N excluded) for infants ≥1500 g birth weight (point estimates and 95% confidence interval limits shown). Adjustment is for gestational age, admission day SNAP-II score, and outborn status.

Comparison of Figures 1 and 2 shows that hospitals with low and high incidences of NI among VLBW infants also tended to have lower and higher incidences of NI among larger infants. When sites P and N were excluded from the analysis, site A was also found to have significantly higher incidence of NI than the median hospital for both VLBW and HBW infants. (Figures 3 and 4)

## Discussion

The above results represent data from 17 tertiary NICUs, making up approximately three-quarters of level III neonatal cots in Canada. Canada has publicly-funded health care with a highly regionalized neonatal-perinatal care system in which each tertiary NICU is the referral centre for its geographic region. Consequently, our results approximate a population-based study for three quarters of the Canadian population.

The bivariate analysis suggests that infants who experience at least one episode of nosocomial bacteremia or nosocomial bacterial meningitis are smaller, more preterm, and had higher illness severity on admission. They have lower 5-minute Apgar scores and are more likely to have been transported from another centre. VLBW infants with NI were more likely to have been delivered vaginally. Nosocomial infection rates will vary from institution to institution if these demographic factors vary from region to region. Adjusted data, to some extent, correct for these geographic variations.

Our data show that significant variations in nosocomial infection do occur between units, with crude rates ranging 6.7% to 74.5% among VLBW infants, and 0.1% to 17.0% among HBW infants. These variations persist after adjustment for gestation, outborn status and admission illness severity. These findings are not unique to hospital acquired infections: using data from the same population of infants, other authors have identified between-site variations in the crude and adjusted rates of both mortality and a number of major neonatal morbidities, including chronic lung disease, retinopathy of prematurity, catheter-related sepsis and intraventricular lesions [6-8,10,18]. There seems little doubt that these differences in outcome exist, the important question remains as to the underlying reasons for the differences.

Multivariate analysis suggests that, in addition to gestation and acuity, a number of therapeutic interventions are associated with nosocomial infection. These include mechanical ventilation, vascular access (central and peripheral), and parenteral nutrition. These factors are predictive in cohorts of both VLBW and HBW babies – supporting their place as clinically significant risks factors for NI. The preponderance of NI among HBW babies with congenital anomalies may reflect surgical interventions, or the possibility of immunocompromise. We recognize that there are likely to be other, unrecorded demographic factors or interventions, that may account for risk, such as feeding practices or ethnicity – these remain unaccounted for in our study.

There is little doubt from this study that nosocomial infection is associated with significant in-hospital morbidity, but the results on mortality are more subtle. Unadjusted mortality in VLBW infants was not increased by an episode of nosocomial sepsis. A possible explanation is that the effect size is small because most deaths among VLBW infants occur in the first few days of life from other causes, for example cardiorespiratory failure, whereas NI occurs more commonly at a later age. In addition, coagulase negative staphylococcal infections (the commonest isolate in NICU) are reported to have a relatively low mortality in VLBW infants (2% or less in three large cohort studies [19-21]), making a small contribution to overall mortality that may not achieve statistical significance.

Between-site analysis confirmed that inter-NICU variation in NI rates persisted after risk adjustment for birth weight, gestation and illness severity in VLBW infants. The 3 sites with lower than median incidence of NI for VLBW infants, also had a lower incidence of NI for HBW infants. Three out of 4 sites with higher than median incidence of NI for HBW infants also had higher incidence of NI for VLBW infants. The impression that overall site performance is consistent for infants at different birth weight groups supports the contention that factors other that those recorded and adjusted for in this study, probably related to interventions or the NICU environment, contribute to the risk of nosocomial infection – especially in VLBW infants, with whom most of the burden of NI lies.

We have seen that nosocomial infection rates vary across Canada and are significantly affected by clinical interventions. However, a number of important factors have not been adjusted for, particularly those related to organism virulence or infectivity, and population susceptibility. One could hypothesize that organism virulence or infectivity varies across the country, as does the susceptibility of population groups. There is some evidence to support variation in genetic susceptibility: Ahrens et al [22] reported that VLBW neonates with specific CD14 gene mutations had a greater rate of neonatal sepsis than those without. A more detailed study of population demographics and organism subtypes may reveal whether these factors impact on nosocomial infection rates. Similarly, environmental factors such as unit layout and clinical practices may affect nosocomial infection rates – the extensive list of potential confounders may require an alternative research methodology to discover association and/or causation. Bloom et al [23] reported decreased rates of nosocomial infections by sharing information about differences between NICUs with high and low nosocomial infection rates, and addressing these variations in clinical practice. The Canadian Neonatal Network is embarking on a multicentre study to examine the effect of multiple evidence-based practice interventions on nosocomial infection rates, which will hopefully answer some of these questions.

A limitation of our study is that it is possible that some positive blood or CSF cultures are in fact false positives. Struthers et al [24] estimated that 5% of positive blood cultures were false, suggesting that the vast majority of confirmed bacteremia is true. However, it might be argued that even false positive results represent a significant burden of illness, and including these episodes may fairly represent this burden. Another limitation of this type of observational study is the difficulty in differentiating cause and effect. The observational data in this study were presented using univariate, unadjusted and risk-adjusted multivariate models. Unfortunately, these analyses cannot account for the temporal relationships between events, nor do they adjust for risk factors that are not included in data collection. However, the strengths of the logistic regression model are its robustness and extensive use in similar studies. Future studies should take temporal relationships into account, permitting time-sensitive analysis, better inference of cause and effect, and assessment of attributable risk.

## Conclusion

Rates of nosocomial infection in Canadian NICUs vary considerably, even after correction for known risk factors. The data suggest that this variation is due, in part, to differences in clinical practices, and therefore may be amenable to interventions that alter practice. More study, and alternative study designs, may be required to evaluate contributing factors and effect practice change.

## Canadian Neonatal Network

Shoo K. Lee (Coordinator, Canadian Neonatal Network; Centre for Healthcare Innovation and Improvement, Vancouver, BC); Wayne Andrews (Charles A. Janeway Child Health Centre, St John's, NF); Ranjit Baboolal (North York Hospital, N. York, ON); Jill Boulton (St Joseph's Health Centre, London, ON; previously Mt Sinai Hospital, Toronto, ON); David Brabyn (Royal Columbian Hospital, New Westminster, BC); David S.C. Lee (St Joseph's Health Centre; London, ON); Derek Matthew (Victoria General Hospital (Victoria, BC); Douglas D. McMillan (IWK-Grace Health Centre for Women, Children and Families, Halifax, NS; formerly Foothills Hospital, Calgary, AB); Christine Newman (Hospital for Sick Children; Toronto, ON); Arne Ohlsson (Mt Sinai Hospital, Toronto, ON; formerly Women's College Hospital, Toronto, ON); Abraham Peliowski (Royal Alexandra Hospital, Edmonton, AB); Margaret Pendray (Children's & Women's Health Centre of British Columbia (Vancouver, BC); Koravangattu Sankaran (Royal University Hospital, Saskatoon, SK); Barbara Schmidt (Hamilton Health Sciences Corporation, Hamilton, ON); Mary Seshia (Health Sciences Centre, Winnipeg, MB); Anne Synnes (Children's and Women's Health Centre of British Columbia, Vancouver, BC; formerly Montreal Children's Hospital, Montreal, PQ); Paul Thiessen (Children's & Women's Health Centre of British Columbia, Vancouver, BC); Robin Walker (Children's Hospital of Eastern Ontario and Ottawa General Hospital, Ottawa, ON); Robin Whyte (IWK-Grace Health Centre for Women, Children and Families, Halifax, NS).

### Coordinating centre staff

Holly Bavinton; Stella Karuri; Zhenguo Qiu; Sarka Lisonkova, Shawn Stewart.

## Abbreviations

Nosocomial infection (NI)

Neonatal Intensive Care Unit (NICU)

Score for Neonatal Acute Physiology, Version II (SNAP-II)

Neonatal Therapeutic Intensity Scoring System (NTISS)

Very low birth weight (VLBW)

Higher birth weight (HBW)

Cerebrospinal fluid (CSF)

Standard deviation (SD)

Confidence interval (CI)

## Competing interests

The author(s) declare that they have no competing interests.

## Authors' contributions

KA interpreted data and drafted the manuscript. DDM, WA and MP were site investigators. ZQ performed statistical analysis and interpretation. SKL was the principal investigator and drafted the manuscript. All these individuals read and approved the final manuscript. The CNN represents all site investigators, and was responsible for organization and administration of the SNAP study, and subsequent data flow.

## Pre-publication history

The pre-publication history for this paper can be accessed here:


